# Ultrasensitive, Label-Free Voltammetric Detection of Dibutyl Phthalate Based on Poly-l-lysine/poly(3,4-ethylenedioxythiophene)-porous Graphene Nanocomposite and Molecularly Imprinted Polymers

**DOI:** 10.3390/bios14030121

**Published:** 2024-02-23

**Authors:** Chuanxiang Zhang, Song Li, Lingxiao Tang, Shuo Li, Changchun Hu, Dan Zhang, Long Chao, Xueying Liu, Yimin Tan, Yan Deng

**Affiliations:** 1 College of Packing and Materials Engineering, Hunan University of Technology, Zhuzhou 412007, China; 2 Hunan Key Laboratory of Biomedical Nanomaterials and Devices, College of Life Science and Chemistry, Hunan University of Technology, Zhuzhou 412007, China

**Keywords:** molecular imprinting polymers (MIP), poly(3,4-ethylenedioxythiophene), porous graphene, electrochemical sensor, dibutyl phthalate

## Abstract

Development of an efficient technique for accurate and sensitive dibutyl phthalate (DBP) determination is crucial for food safety and environment protection. An ultrasensitive molecularly imprinted polymers (MIP) voltammetric sensor was herein engineered for the specific determination of DBP using poly-l-lysine/poly(3,4-ethylenedioxythiophene)/porous graphene nanocomposite (PLL/PEDOT−PG) and poly(o-phenylenediamine)-imprinted film as a label-free and sensing platform. Fabrication of PEDOT−PG nanocomposites was achieved through a simple liquid–liquid interfacial polymerization. Subsequently, poly-l-lysine (PLL) functionalization was employed to enhance the dispersibility and stability of the prepared PEDOT−PG, as well as promote its adhesion on the sensor surface. In the presence of DBP, the imprinted poly(o-phenylenediamine) film was formed on the surface of PLL/PEDOT−PG. Investigation of the physical properties and electrochemical behavior of the MIP/PLL/PEDOT−PG indicates that the incorporation of PG into PEDOT, with PLL uniformly wrapping its surface, significantly enhanced conductivity, carrier mobility, stability, and provided a larger surface area for specific recognition sites. Under optimal experimental conditions, the electrochemical response exhibited a linear relationship with a logarithm of DBP concentration within the range of 1 fM to 5 µM, with the detection limit as low as 0.88 fM. The method demonstrated exceptional stability and repeatability and has been successfully applied to quantify DBP in plastic packaging materials.

## 1. Introduction

Dibutyl phthalate (DBP) is widely used as a plasticizer in various applications, such as food packaging, medical equipment, printing ink, cosmetics, and adhesives [1,2,3,4]. It enhances the flexibility, durability, and transparency of polymers like PVC [5,6]. However, due to weak hydrogen bonds or van der Waals forces between DBP molecules and polymer carbon chains instead of chemical bonds, DBP can easily be released into the environment and contaminate water and food through external factors [7]. Being an endocrine disruptor with long-time stability and resistance to degradation [8,9], DBP can be enriched through the food chain and ultimately endanger human health [10]. A large number of toxicological studies have shown that DBP exhibits chronic and acute toxicity by adversely affecting reproductive development and the endocrine system in organisms. It also leads to metabolic disorders, malformations, cancers, and genetic mutations of organisms [11,12,13,14]. By recognizing its hazardous nature, the US Environmental Protection Agency (US-EPA) has listed DBP as a priority pollutant [15,16]. Therefore, it is urgent to design a sensitive and accurate trace detection system for DBP. Currently available methods for DBP detection include high-performance liquid chromatography (HPLC) [17], gas chromatography–mass spectrometry (GCMS) [18,19], high-performance liquid chromatography–mass spectrometry (HPLC−MS) [20,21], and enzyme-linked immunosorbent assay (ELISA) [22,23,24]. Nevertheless, these analytical methods have limitations; for instance, complex sample pretreatment, expensive detection equipment, and professional operators. In comparison, electrochemical sensors are considered as a sensitive and fast analysis method with simple equipment and an efficient detection time [25,26].

However, as a member of phthalates (PAEs), DBP shares a similar molecular structure with homologues such as DOP, DEHP, DEP, etc. Traditional electrochemical sensors are unable to specifically identify DBP. However, the combination of molecular imprinting technology and electrochemical sensors can achieve enhanced sensitivity, specificity, simplicity, and the rapid detection of target molecules [27]. Molecular imprinting technology involves polymerizing the template molecule with the appropriate functional monomer to obtain a polymer, and then the template molecule is eluted by a certain method to create cavities and re-recognition sites within the polymer that are complementary to the template molecular space. Through these cavities and re-recognition sites, accurate and quick identification of the target molecules becomes possible by specifically recognizing the template molecules [28,29]. Nevertheless, most molecularly imprinted polymers lack conductivity, which significantly affects the transfer of electrons on the electrode surface, thereby reducing the detection sensitivity. Utilizing nanomaterials for modifying electrode surfaces proves effective in improving adhesion of MIP to the electrode and enhancing the sensitivity of electrochemical analysis [30,31]. Studies have shown that the electrochemical sensors modified by nanomaterials have satisfactory sensitivity and detection limit [32,33].

Porous graphene (PG) is a new derivative of graphene, which is a carbon material with a nanoporous structure on both sides of the surface of graphene. The pores in the porous graphite are vacancies resulting from the removal or transfer of carbon atoms from the lattice to the surface, which can be considered as defects. The introduction of pores effectively opens the energy band gap of graphene and expands its application range in electronic devices. PG has dual advantages as it retains the excellent properties of graphene while also increasing its specific surface area and active sites by pore structure compared to the surface of two-dimensional graphene. This enhancement accelerates the rate of mass transport, and its atomic-level pores play a role in screening ions/molecules of different sizes [34,35]. Furthermore, it has been reported that, the combination of graphene materials with conductive polymer composites can address issues such as poor disperse and low electrical conductivity typically associated with pristine graphenes. This combination strengthens the synergistic effects between individual components and obtains improved properties, including high conductivity, large surface area, rapid electron transfer, excellent mechanical properties, and superior biocompatibility [36,37]. Among the various conductive polymers investigated so far, poly(3,4-ethylenedioxythiophene) (PEDOT) stands out due to its high conductivity and extremely narrow band gap within the visible range along with excellent optical transparency, good electrochromic properties, and long-term electrochemical stability [38]. The presence of special ethylenedioxy bridges at the 3- and 4-positions of thiophene not only reduces the oxidation potential but also prevents the unwanted α-β and β-β coupling in the polymer backbones, resulting in high regioregularity [39]. Li et al. [40] prepared a graphene oxide doped PEDOT film by the electrodeposition method, which was used for the determination of ascorbic acid, dopamine, and uric acid. The prepared PEDOT−GO nanocomposites are endowed with a special structure and high specific surface area. The electrochemical sensor exhibits good stability and acceptable sensitivity. Zuo et al. [41] prepared PEDOT−GO nanocomposites by a simple liquid–liquid interfacial polymerization method. A PEDOT−GO modified glassy carbon electrode (GCE) showed high electrocatalytic activity for Hg^2+^, good selectivity, a wide linear range, and good long-term stability.

It is precisely because of its unique structure, on the one hand, that the introduction of inorganic material (PG) can adjust the polymerization structure of PEDOT, improve its carrier mobility, and expand the specific surface area during the synthesis of PG and PEDOT composites. In addition, the polymerization of EDOT can form a conductive film on the surface of PG, thereby improving the electroconductivity and stability of the composite [42,43]. Poly-l-lysine (PLL) is a cationic polymer containing a variety of amino groups. Many aminobutyl groups within PLL have both the positive charge (−NH^3+^) from primary amines and hydrophobic properties from butyl spacers between primary amines. Therefore, using PLL for the surface functionalization of materials not only increases the conductivity of materials in aqueous solution, but also enhances dispersion and stability [44,45]. In addition, PLL itself has a certain viscosity, which can increase adhesion of the composite material onto the electrode surface [46].

**Scheme 1:**
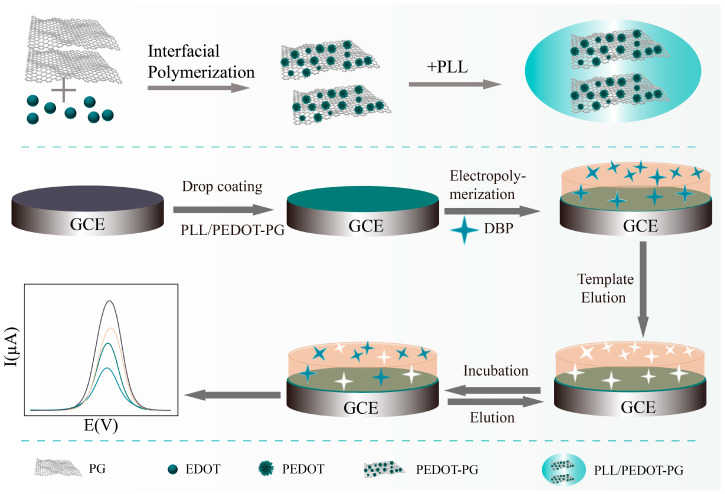
Schematic illustration for preparation of MIP/PLL/PEDOT−PG/GCE and electrochemical determination of DBP.

In this study, PEDOT−PG composites were prepared by means of liquid–liquid interface polymerization, dispersing EDOT monomer, and PG in the organic phase and aqueous phase, respectively. Then, the prepared PEDOT−PG was functionalized with PLL, so that PLL/PEDOT−PG had good dispersion, stability, and adhesion on the sensor surface. Furthermore, using o-phenylenediamine (o-PD) as a monomer and DBP as a template molecule, the DBP molecular-imprinted polymer membranes was electropolymerized onto the PLL/PEDOT−PG/GCE surface by cyclic voltammetry electropolymerization (Scheme 1). This sensor can detect DBP with high sensitivity and selectivity, and be used for the detection of DBP in real samples.

## 2. Experimental

### 2.1. Chemicals and Reagents

Graphene was obtained from XFNANO (Nanjing, China). Chloroform (CHCl_3_), glacial acetic acid, methyl alcohol, cyclohexane, ferric chloride (FeCl_3_), Potassium erricyanide (K_3_[Fe(CN)_6_]), and Potassium chloride (KCl) were acquired from Sinophram Chemical Reagents Co., Ltd. (Shanghai, China). Poly-l-lysine (PLL) and 20 × PBS buffer were purchased from Sangon Biotech Co., Ltd. (Shanghai, China). 3,4-ethylenedioxythiophene (EDOT), o-Phenylenediamine(o-PD), Dibutyl phthalate (DBP), Dimethyl phthalate (DMP), Diethyl phthalate (DEP), Dioctyl phthalate (DOP), bis(2-ethylhexyl) phthalate (DEHP), and Diisononyl phthalate (DINP) were purchased from Aladdin Regents (Shanghai, China).

### 2.2. Apparatus

Scanning electron microscopy (SEM) images and energy dispersive spectroscopy (EDS) were obtained from a Sigma 300 scanning electron microscope (ZEISS, Oberkohen, Germany). Transmission electron micrographs (TEM) were collected using a Jem-2100F (JEOL Co., Ltd., Tokyo, Japan). Raman spectra were measured by HORIBA HR Evolution laser Raman spectrometer (HORIBA, PAR, France). FT-IR spectra were recorded on a Thermo Scientific Nicolet iS20 ATR-FTIR instrument (Thermo, MA, USA). X-ray photoelectron spectroscopy (XPS) measurements were carried out by a Thermo Scientific K-Alpha (Thermo, MA, USA). The electrochemical measurements were conducted on a PGST AT302N Electrochemical Workstation (Metrohm, Herisau, Switzerland).

### 2.3. Preparation of PLL/PEDOT−PG Nanocomposite

#### 2.3.1. Preparation of PG

The PG was prepared by impregnating 50 mg graphene in 100 mL 0.5 mol/L KOH solution, stirring for 2 h, then ultrasonic for 1 h, and standing for 12 h. The mixture of graphene and KOH solution was transferred to a hydrothermal kettle, which was heated to 140 °C in the oven for 2 h, and then removed and cooled to room temperature. The mixture obtained by the above reaction was subjected to suction filtration to remove the remaining lye, and then washed with 3 wt% dilute hydrochloric acid for 5 min. Finally, the filtrate was washed with distilled water to neutral, suction filtered, and the sample was dried at 40 °C in a blast drying oven for 5 h to obtain a porous graphene product.

#### 2.3.2. Synthesis of PLL/PEDOT−PG

PEDOT−PG nanocomposites were prepared by liquid–liquid interface polymerization according to previous literature [41]. In a typical procedure, 1 mL of PG (2.0 mg·mL^−1^) dispersion was added to 1 mL of FeCl_3_ (1 mol·mL^−1^) solution, in which FeCl_3_ acted as an oxidant. After ultrasonic treatment for 10 min, the mixture was added dropwise into 2 mL of CHCl_3_ solution containing EDOT (25 mg·mL^−1^) to create an oil and water separation interface between the two layers. The mixture reacts statically in a water bath at 50 °C for 12 h. Centrifuging the upper mixture, the precipitate was washed twice with ethanol and double distilled water, respectively. The collected nanocomposites were further tested. The washed PEDOT−PG complex was dispersed with 1 mL deionized water, and the dispersion solution was mixed with poly-lysine (PLL) at 3:2 (*v*:*v*). The dispersion solution was shaken for 2 h in a shaking bed, and then placed in a refrigerator at 4 °C for full reaction of the PLL and PEDOT−PG complex through non-covalent bonding.

### 2.4. Preparation of Molecularly Imprinted Electrode (MIP/PLL/PEDOT−PG/GCE)

Prior to modification, the GCE was polished with aluminum slurry until a mirror surface was obtained. Then, it was rinsed thoroughly with absolute alcohol and water in an ultrasonic bath successively. Scheme 1 displayed the stepwise fabrication of the MIP/PLL/PEDOT−PG/GCE. First, 5 μL of PLL/PEDOT−PG suspensions were dropped onto the GCE and dried at 25 °C. After that, the prepared PLL/PEDOT−PG/GCE was immersed in a 25 mL PBS electrolyte (0.01 mM, pH = 7.4) containing 5 mM o-PD and 2 mM DBP, and 12 consecutive potentiodynamic cycles were performed in the potential range 0.2–0.8 V at a scan rate of 50 mv·s^−1^ to yield a DBP imprinted o-PD film. After the polymerization was completed, the electrode was washed with deionized water to remove the unpolymerized o-PD on the surface and dried. At room temperature, the MIP/PLL/PEDOT−PG/GCE was obtained by immersing the electrode in a mixed solution of methanol/20% acetic acid (1:10, *v*:*v*) for 30 min and washing off the template molecule DBP. As a comparative experiment, the non-molecularly imprinted polymers (NIPs) modified electrode was prepared in the same way, except that DBP was not added during the electropolymerization process.

### 2.5. Electrochemical Measurements

MIP/PLL/PEDOT−PG/GCE was initially incubated in PBS solution (pH = 5) containing DBP of different concentrations for 15 min under magnetic stirring. The MIP/PLL/PEDOT−PG/GCE was then transferred into 5 mmol L^−1^ K_3_ [Fe(CN)_6_] and 0.1 mol L^−1^ KCl electrolytes for differential pulse voltammetry (DPV) measurements.

### 2.6. Detection of DBP in Real Samples

Two types of PVC plastic wrap and PET plastic bottles were selected as actual samples to measure the DBP contained in them. PVC plastic wrap was purchased from the local supermarket, and PET plastic bottles for a certain brand of mineral water bottles were used. The above materials were washed, dried, cut into small pieces of less than 2 mm × 2 mm, about 1.0 g each, and 0.2 g was weighed and dispersed in 50 mL water and 50 mL C_6_H_12_ for 5 days to make six samples, named PVC1-W (Water), PVC1-C (C_6_H_12_), PVC2-W, PVC2-C, PET-W, and PET-C. The extract was filtered with a 0.22 μm filter membrane, dried with argon, and diluted with ethanol. Different concentrations of DBP standard solution were added to the six groups of sample solutions, and the recovery rate was determined.

## 3. Results and Discussion

### 3.1. Characterization of PLL/PEDOT−PG Composite

**Figure 1:**
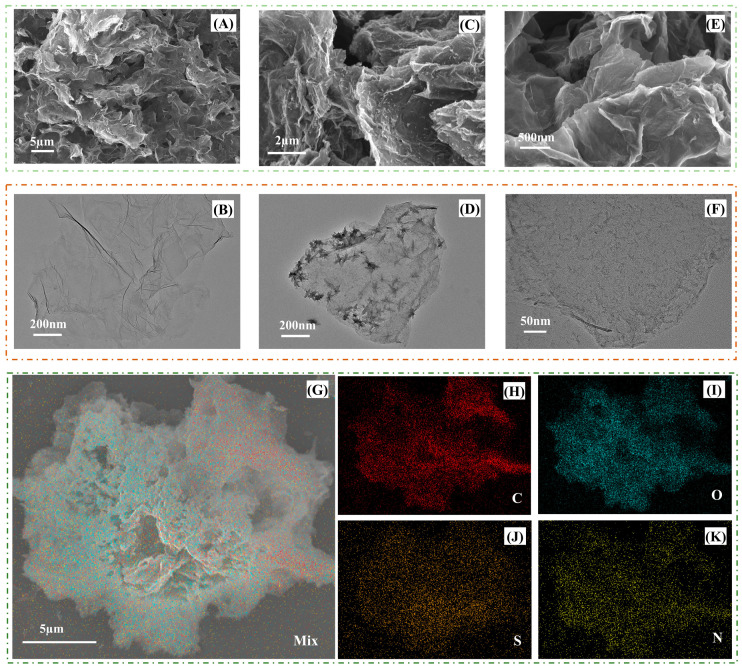
SEM images of (**A**) PG, (**C**) PEDOT−PG, (**E**) PLL/PEDOT−PG; TEM images of (**B**) PG and (**D**) PEDOT−PG; (**F**) PLL/PEDOT−PG, Elemental mapping images of Mix (**G**), C (**H**), O (**I**), S (**J**), N (**K**) in PLL/PEDOT−PG composite.

The structure and morphology of PG, PEDOT−PG, and PLL/PEDOT−PG were characterized by SEM, TEM, and EDS. Figure 1A,B are the SEM and TEM images of porous graphene, respectively. Compared with the smooth surface structure of graphene, it was seen that the prepared PG had a uniform three-dimensional cross-linked network pore structure, indicating that KOH had effectively etched graphene. The porous structure was not only conducive to increasing the specific surface area of graphene, providing more active sites, but also accelerating the diffusion and transfer of ions, which is very conducive to applications in electrochemical sensors. Figure 1C,D are the SEM and TEM images of the prepared PEDOT−PG, respectively. It was seen that a large number of PEDOT nanorod particles uniformly cover the surface of PG. The π–π interaction and van der Waals interaction between the aromatic ring of PG and PEDOT play a significant role in the formation of PEDOT−PG composites [47,48]. The diameter and length of PEDOT nanorods were between 20–30 nm and 100–200 nm, respectively, which further effectively expanded the surface area of PG. Figure 1E, F are the SEM and TEM images of the prepared PLL/PEDOT−PG, respectively. We can see that the addition of PLL did not change the morphology of PEDOT−PG, but only added a layer of film-like material to cover the surface of PEDOT−PG. The uniform distribution of C, S, O, and N elements in the DES energy spectrum of PLL/PEDOT−PG (Figure 1G–K) further illustrated the formation of PEDOT nanorods on the surface of PG. PLL was uniformly wrapped on the surface of PEDOT−PG, which also means that PLL/PEDOT−PG was successfully prepared.

**Figure 2:**
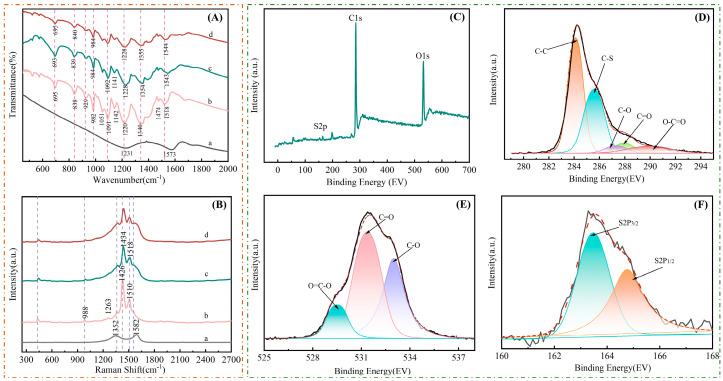
(**A**) Infrared spectra of and (**B**) Raman spectra of (a) PG, (b) PEDOT, (c) PEDOT−PG, and (d) PLL/PEDOT−PG. (**C**) XPS survey spectra of PEDOT−PG. High resolution (**D**) C1s, (**E**) O1s, (**F**) S2p XPS spectra of PEDOT−PG.

Figure 2A gives the FT-IR spectrum of PG, PEDOT, PEDOT−PG, and PLL/PEDOT−PG. The band at 1340 cm^−1^ (spectrum (b)), which was due to C−C and C=C stretching of the quinoidal structure from the thiophene ring, shifts to 1354 cm^−1^ in spectrum (c,d). The blue-shift of this band indicates that the conjugated chain of PEDOT is doped with PG as a macromolecule [49,50]. A broad absorption band at 1518 cm^−1^ in the spectrum (b) is attributed to the C=C bond. This band’s position is determined by the doping level of the polymer [51], and it shifts to 1543 cm^−1^. This indicates that PG acted as a dopant for conductive PEDOT in the polymer chain. Compared with PEDOT−PG, the infrared spectrum of PLL/PEDOT−PG has no significant change, indicating that there was no chemical reaction between PLL and PEDOT−PG.

The Raman spectra of PG, PEDOT, PEDOT−PG, and PLL/PEDOT−PG are shown in Figure 2B. It can be seen from the curve (a) that PG has a characteristic absorption at about 1352 cm^−1^ and 1582 cm^−1^, which were attributed to the D and G bands of PG, respectively. In general, the G band is the characteristic peak for sp2 hybrid carbon, and the D band was related to the defects inside the graphene, indicating that the crystal structure of graphite was destroyed during the activation of graphene by KOH, resulting in lattice defects. It can be seen that the samples (curve (b)) exhibited strong absorption at 1426 cm^−1^ and 1510 cm^−1^, and the absorption peaks correspond to the stretching vibration of the symmetric Cα=Cβ(-O) bond and asymmetric C=C bond on the conjugated thiophene ring. The peaks at 1263 cm^−1^ and 988 cm^−1^ are due to the inter-ring stretching and oxyethylene ring deformation absorption of PEDOT segments [48]. Although the overall Raman spectrum of the composite PEDOT−PG (curve (c)) was similar to that of PEDOT, its characteristic peak position was different from that of PEDOT because of the addition of PG material. It can be clearly distinguished that the peaks belonging to the C=C bond in the composite showed different degrees of blueshift. The absorption peaks at 1426 cm^−1^ and 1510 cm^−1^ in PEDOT were shifted to 1434 cm^−1^ and 1518 cm^−1^ in the PEDOT−PG composites, respectively. Therefore, we can speculate that there may be a strong interaction π–π bond between PG and PEDOT molecules, which is consistent with the relevant literature [52,53]. Comparing the Raman spectra of PEDOT−PG and PLL/PEDOT−PG (curve (d)), there was no obvious change, which further indicates that there was no chemical reaction between PLL and PEDOT−PG.

The composition of the prepared PEDOT−PG was characterized by XPS. Figure 2C shows that the wide scan XPS spectrum of PEDOT−PG indicated the existence of C, O, and S. The high-resolution XPS spectra for C1s, O1s, and S2p in the PEDOT−PG samples are displayed in Figure 2D–F. Figure 2D presents the C1s spectra for PEDOT−PG, the peaks of C1s at 284.3, 285.8, and 287.2 eV, associated to the C-C, C-S, and C-O bond, respectively. The two peaks at 287.5 and 289.8 eV should belong to the C=O and O=C-O groups in PG, indicating that PG was mildly oxidized during etching. Figure 2E exhibits the O1s XPS spectrum for PEDOT−PG. The peaks at 532.9, 531.6, and 530.5 eV associated to the C-O groups in PEDOT−PG, C=O, and O=C-O groups in PG, respectively. The S2p spectrum for PEDOT−PG (Figure 2F) showed the characteristic peaks for spin-splitting double state S2p at 163.4 (S2p_3/2_) and 164.6 eV (S2p_1/2_), which belong to the thiophene ring in PEDOT [54]. The XPS results showed that PEDOT−PG was successfully synthesized.

**Figure 3:**
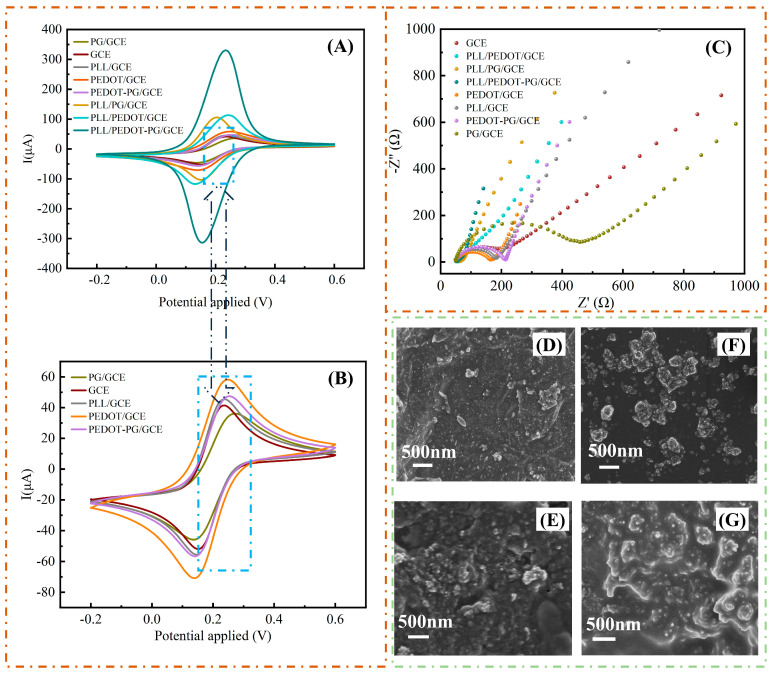
(**A**,**B**) CV plots and (**C**) EIS plots for GCE, PG/GCE, PLL/GCE, PEDOT/GCE, PLL/PG/GCE, PLL/PEDOT/GCE, PLL/PEDOT−PG/GCE, SEM images of MIP/PLL/PEDOT−PG/GCE before (**D**) and after (**E**) template elution, NIP/PLL/PEDOT−PG/GCE before (**F**) and after (**G**) elution.

To evaluate the electrochemical performance of prepared PLL/PEDOT−PG/GCE, the electrochemical behavior of GCE modified by PG, PEDOT, PEDOT−PG, PLL, PLL/PG, PLL/PEDOT, PLL/PEDOT−PG at the same concentration was studied by CV and EIS. As can be seen from Figure 3A,B, the current value did not increase significantly compared with that of GCE after the PLL was modified on the electrode, which proves that the PLL had dispersion, coating, and adhesion effects on the composite material, and had no obvious improvement on the electrochemical signal. After the PG was modified on the electrode, the current signal was smaller than that of GCE. The reason was that the conjugated structure of graphene gives it relative electronegativity, which excludes [Fe(CN)_6_]^3−^ in the solution and hinders the electron transfer on the electrode surface, resulting in a low current peak. The current value of PLL/PG/GCE is greatly improved compared with GCE, because the coating of PLL can eliminate electrostatic repulsion. The electrochemical signal value for PEDOT−PG modified on the electrode was higher than that of PG/GCE, but was still very low, which proved that the electronegativity of PG still existed. Compared with PLL/PG/GCE, PLL/PEDOT−PG/GCE showed higher peak current, faster electron transfer rate, and better conductivity.

To further characterize the electrochemical properties of PLL/PEDOT−PG/GCE, EIS was used to investigate the change of electrode surface state (Figure 3C). Compared with GCE, PG/GCE, PEDOT/GCE, PLL/GCE, PEDOT−PG/GCE, PLL/PG/GCE, PLL/PEDOT/GCE, the arc of PLL/PEDOT−PG/GCE in the high frequency region is very small, mainly based on the linear development in the low frequency region, indicating that its charge transfer impedance is very small, and the electrode process is almost not controlled by charge transfer, but mainly controlled by diffusion process. The slope of the impedance diagram for PLL/PEDOT−PG/GCE is the largest in the low frequency region, indicating that the impedance value of [Fe(CN)_6_]^3−^ diffusion at the interface of PLL/PEDOT−PG/was smallest, and the prepared PLL/PEDOT−PG composite had excellent electrical conductivity.

The reason is that the doping of porous graphene in the conductive polymer PEDOT expands the specific surface area of the composite material, improves the carrier mobility, and has higher conductivity and better stability. In addition, the synergistic effect of PEDOT and PG plays an important role in signal enhancement [55].

### 3.2. Characterization of MIP/PLL/PEDOT−PG/GCE

SEM was applied to characterize the sensor surface of MIP/PLL/PEDOT−PG/GCE before (Figure 3D) and after template elution (Figure 3E), and NIP/PLL/PEDOT−PG/GCE before (Figure 3F) and after applying the same washing protocol (Figure 3G). Before eluting the template, the MIP/PLL/PEDOT−PG/GCE was covered by a uniformly arranged, dense poly-o-phenylenediamine membrane (PPD) (Figure 3D). After the washing step, a rougher surface can be observed in Figure 3E, which indicates the formation of a more porous film. In contrast, the surface of the NIP/PLL/PE-DOT−PG/GCE was very dense, rough, and non-uniform, with significant aggregation (Figure 3F). This may be related to the polymerization mechanism of o-PD; the absence of template molecules greatly accelerates the electropolymerization of o-PD and is prone to particle aggregation. Similar to the MIP, the porosity of the NIP/PLL/PEDOT−PG/GCE was also increased, although to a lesser extent, after washing (Figure 3G), demonstrating an effect of the washing procedure on the morphology of the films.

**Figure 4:**
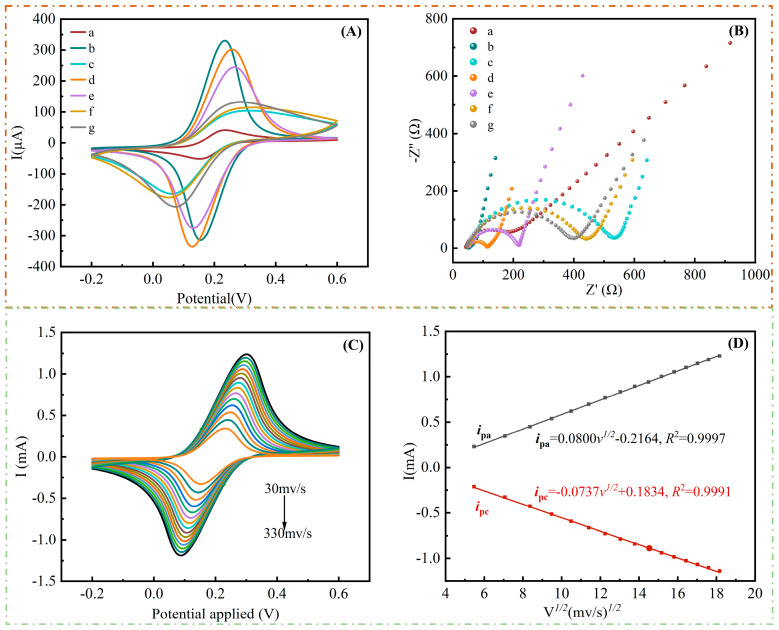
(**A**) CV plots and (**B**) EIS plots of GCE (a), PLL/PEDOT−PG/GCE (b), MIP/PLL/PEDOT−PG/GCE (c, Before Elution), MIP/PLL/PEDOT−PG/GCE (d), MIP/PLL/PEDOT−PG/GCE (e, After incubation), NIP/PLL/PEDOT−PG/GCE (f, Before Elution), NIP/PLL/PEDOT−PG/GCE (g). (**C**) CV plots of MIP/PLL/PEDOT−PG/GCE at different scan rates from 30 mv/s to 330 mv/s in the supporting electrolyte of 10 mM PBS buffer containing 0.1 M KCl and 5 mM K_3_ [Fe(CN)_6_], the frequency range was 10^5^ Hz to 0.1 Hz, the amplitude was 5 mV, and the open circuit potential was 0.203 V, (**D**) Linear variation of I_pa_ and I_pc_ with scan rate.

The stepwise preparation process for MIP/PLL/PEDOT−PG/GCE was studied by cyclic voltammetry (Figure 4A) and EIS (Figure 4B) in an electrolyte with [Fe(CN)_6_]^3−^. Compared with bare GCE (a), the redox peak for the PLL/PEDOT−PG/GCE (b) in the [Fe(CN)_6_]^3−^ electrolyte was greatly improved, and the impedance value (Rct) was much smaller than that of bare GCE, showing that the PLL/PEDOT−PG composite has excellent conductivity. Whereas, after the electropolymerization of o-PD on PLL/PEDOT−PG/GCE(c), the CV peaks for the probe almost disappeared, and the Rct value increased significantly, demonstrating the PPD membrane formed by electropolymerization is non-conductive. This PPD films effectively prevented the diffusion of the Fe[(CN)_6_]^3−^ probe to reach the electrode surface, and further hindered electron transfer. The redox peak for [Fe(CN)_6_]^3−^ in MIP/PLL/PEDOT−PG/GCE was significantly improved after removing the template molecule (d), which indicated that the DBP molecule had been effectively removed from the imprinted cavities during the elution process, leaving some holes on the surface of the molecularly imprinted membrane, so that the [Fe(CN)_6_]^3−^ could enter the cavity and showed an obvious redox peak. The recognition sites contained in these imprinted holes matched the size, shape, space, and configuration of DBP molecules. After the MIP/PLL/PEDOT−PG/GCE was re-enriched with DBP, DBP specifically re-occupied the holes, reducing the redox peak, which indicates that the DBP molecules were bound to the imprinted cavities and hindered the transfer of electrons in the electrolyte solution (e). NIP/PLL/PEDOT−PG/GCE showed no significant change in K_3_[Fe(CN)_6_] electrical signals after polymerization (f) and elution (g), indicating that no imprinted holes were produced on the electrode surface, which indicated the successful preparation of MIP/PLL/PEDOT−PG/GCE. Moreover, the cyclic voltammetric curve of MIP/PLL/PEDOT−PG/GCE is shown in Figure 4C. It can be observed that the peak current increased gradually with increased scanning rate. The anodic peak current (I_pa_) and cathodic peak current (I_pc_) showed a satisfactory linear relationship with v1/2 (v is the scanning rate) (Figure 4D). The equations were I_pa_ = 0.0800v^1/2^ − 0.2164 (R^2^ = 0.9997) and I_pc_ = −0.0737v^1/2^ + 0.1834 (R^2^ = 0.9991), respectively, indicating that the electrochemical responses of [Fe(CN)_6_]^3−^ on MIP/PLL/PEDOT−PG/GCE was a diffusion-controlled process [56].

It can be observed from the above construction process for molecular-imprinted sensors that the Nyquist diagram for each sensor was consistent with the CV diagram characterization results, indicating that the prepared MIP/PLL/PEDOT−PG/GCE was successfully constructed and can be applied in the detection of DBP.

### 3.3. Optimization of Preparation Conditions for MIP/PLL/PEDOT−PG/GCE

#### 3.3.1. The Ratio of o-PD to DBP

**Figure 5:**
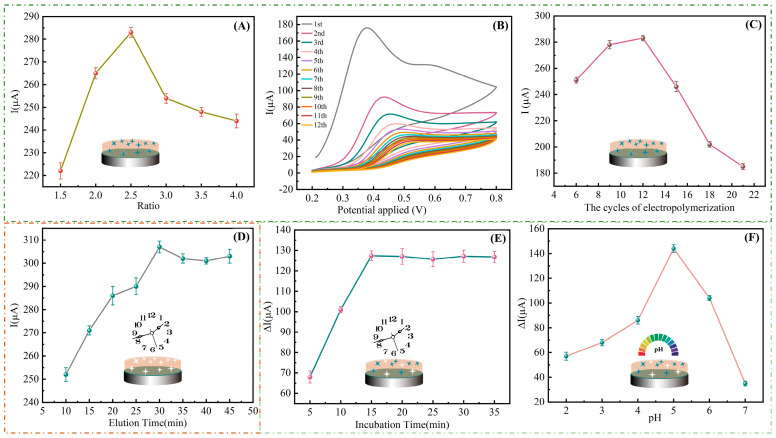
Optimization of analytical parameters. o−PD: DBP molar ratio (**A**), The electro−polymerization curve of o−PD and DBP on PLL/PEDOT−PG/GCE (**B**), electropolymerization cycles (**C**), elution time on the elution efficiency of template molecules (**D**), effect of incubation time on the ΔI of DBP (**E**), plots of the ΔI of DBP against pH values (**F**).

To generate the imprinted PPD film on the surface of PLL/PEDOT−PG/GCE, the electropolymerization of o-PD was carried out in the presence of DBP. Mass transfer occurs in the polymerization solution through the diffusion control process without external force. Through cyclic voltammetry, monomer o-PD was electropolymerized on the surface of PLL/PEDOT−PG/GCE, and carbonyl group in the DBP molecule combined with an amino group in the o-PD molecule was due to the formation of s hydrogen bond. Therefore, the DBP molecule was embedded in the polymerization film and deposited on the electrode surface during the electro-polymerization of o-PD. The ratio of o-PD to the DBP had a great influence on the number and effect of the molecularly imprinted polymer recognition sites. Therefore, it is necessary to optimize the ratio of the functional monomer o-PD and template molecule DBP in the solution. This was conducted by keeping the o-PD concentration at 5 mM constant and varying the concentration of DBP in the solution with o-PD:DBP concentration ratios of 1.5:1, 2.0:1, 2.5:1, 3.0:1, 3.5:1, and 4.0:1. Using cyclic voltammetry to form a molecularly imprinted polymer film on the surface of PLL/PEDOT−PG/GCE, the MIP/PLL/PEDOT−PG/GCE (before elution) was obtained. After removing the DBP molecules from the imprinted membrane by elution, the prepared imprinted electrode MIP/PLL/PEDOT−PG/GCE was placed in electrolyte containing [Fe (CN)_6_]^3−^ for DPV detection, to determine the optimal ratio of o-PD and DBP through peak changes. From Figure 5A, it can be seen that, as the ratio of the two was increased, the DPV peak showed an increased trend first and then it decreased. The DPV peak reached the highest point at 2.5:1, indicating that when the ratio was less than 2.5, the o-PD content was too low to bind enough template molecules in the polymerization process, resulting in too few binding cavities in the imprinted membrane after elution. Similarly, when the content of o-PD was too high, the removal of DBP molecules from the cross-linked structure was blocked during elution, and it was difficult to form sufficient recognition sites after elution. Therefore, the optimal ratio of o-PD and DBP was set as 2.5:1. 

#### 3.3.2. The Cycles of Electropolymerization

The thickness of the imprinted membrane has influence on the number of recognition sites in the MIP after eluting the template molecules, and affects the response degree for MIP/PLL/PEDOT−PG/GCE to DBP molecules. The thickness of the imprinted film could be reasonably regulated by optimizing the number of electrochemical polymerization cycles. Figure 5B is the polymerization curve for the imprinted membrane. It can be observed that an obvious oxidation peak appeared at about 0.362 V and the peak current was 176.03 μA in the first cycle CV curve for o-PD polymerization, which was due to oxidation of the amino group on o-PD. Since the oxidation of o-PD is completely irreversible, as the number of cyclic voltammetry cycles increases, the oxidation peak current decreases and the peak potential shifts positively, and remained basically unchanged after the 12th cycle. The reason is that o-PD forms a polymer film with poor conductivity on the electrode surface, which inhibits electron transfer. At 12 cycles of polymerization, the peak current value measured is the largest after elution of the imprinting sensor, as shown in Figure 5C This also indicates that the molecular imprinted membrane formed by the polymerization of 12 cycles had the maximum recognition sites for DBP. When the number of polymerization circles was too small, the imprinted membrane with fewer and thinner imprinting sites will be more easily destroyed by elution conditions. Therefore, when the DBP molecules were recombined to the imprinting site, the current peak and charge impedance changed very little. Conversely, a thicker PPD layer is generated when there are too many cycles, which will make it difficult for DBP molecules to be eluted from the polymer, increasing the resistance of the [Fe(CN)_6_]^3−^ probe to the surface of MIP/PLL/PEDOT−PG/GCE, and this leads to the destruction of the sensitivity of the sensor. Therefore, the optimal number of polymerization cycles is 12.

#### 3.3.3. Elution Time

The purpose of solvent elution is to utterly remove the template molecule by destroying the force between the template molecule and the polymer skeleton and extracting the template molecule into the solvent. The choice of solvent should not only consider the solubility of template molecules in solvent, but also take into account the stability of polymerized film in solvent, so as to achieve the effect of removing template molecules without destroying polymer film. As a template elution solvent, methanol has a higher elution efficiency than methylene chloride and acetonitrile because of its larger polarity and better permeability. Adding an appropriate amount of acetic acid to methanol can increase the elution force of the solvent, destroying the binding force between the template and polymer, and effectively reducing the leakage of template molecules. Both acetic acid and methanol are soluble in water, and rinsing the electrode with ultra-pure water after elution can well remove the eluent remaining on the electrode. Therefore, acetic acid and methanol were herein selected as elution solvents. When the elution solvent is too acidic, the spatial structure of the polymer film will be destroyed, making MIP/PLL/PEDOT−PG/GCE unable to adsorb the template molecule DBP again after elution. Finally, it was found that the optimal elution effect was obtained when the volume ratio of methanol to 20% acetic acid was 1:10. The imprinted electrode was placed in an elution solvent and eluted at different times. K_3_ [Fe(CN)_6_] was used for DPV scanning. The optimal elution time was determined according to the peak current. Figure 5D shows that as the elution time increased from 10 min to 45 min, the template molecule DBP gradually dissolved, and the ‘holes’ on the electrode surface increased, while the DPV peak current increased. When the elution time was greater than 30 min, the peak current reduced, which may be due to the slight swelling of the imprinting film caused by prolonged elution and blockage of the imprinting site. This indicates that the DBP molecules were completely elution after 30 min.

#### 3.3.4. Optimization of DBP Detection Conditions

- Incubation time

From Figure 5E, it can be seen that the number of DBP molecules adsorbed by MIP/PLL/PEDOT−PG/GCE gradually increased with increased incubation time, and identification holes were gradually re-filled by DBP, mass transfer channels were closed, oxidation–reduction current intensity was weakened, the peak current value decreased, and peak current difference ΔI (ΔI = I_0_ − I_c_, I_0_, and I_c_ were the current at a DBP concentration of 0 and c, respectively) gradually increased. When the adsorption time exceeded 15 min, the peak current difference ΔI began to remain unchanged, indicating that the PPD film had fast recognition ability and a high affinity for DBP molecules. Therefore, the optimal incubation time was 15 min.

- Buffer pH

Since the buffer pH had an important effect on the sensitivity of the electrochemical sensor, the eluted MIP/PLL/PEDOT−PG/GCE was inserted into dibutyl phthalate PBS buffer at different pH values, and the effect of a pH value from 2.0 to 7.0 on the DPV current response signal was studied. Figure 5F shows the DPV current response on the sensor surface gradually increased as the pH value was increased from 2.0 to 5.0, and as the pH value was further increased, the current response gradually decreased. This indicates that, at a pH of 5.0, the polyo-phenylenediamine with an amino group functions as an amino molecule and readily forms hydrogen bonds with DBP. Consequently, when the buffer solution’s pH is 5, the hydrogen bonding force between the imprinted hole and DBP molecule is at its strongest, resulting in optimal electron transfer capacity for [Fe(CN)_6_]^3−^ on the electrode surface.

#### 3.3.5. Electrochemical Detection of DBP

**Figure 6:**
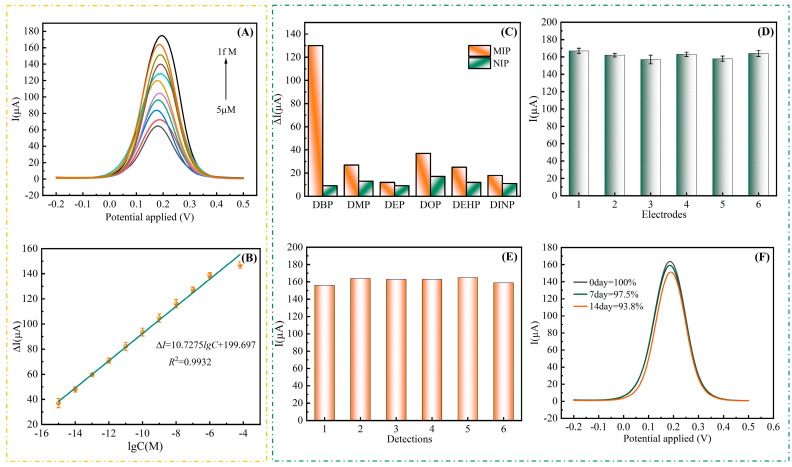
(**A**) DPV of the developed MIP/PLL/PEDOT−PG/GCE sensor to DBP at different concentrations (1 fM, 10 fM, 100 fM, 1 pM, 10 pM, 100 pM, 1 nM, 10 nM, 100 nM, 1 µM, 5 µM) using [Fe(CN)_6_]^3−^ as probe. (**B**) Standard curve between the current response and DBP concentration (R^2^ = 0.9932). (**C**) Selectivity of the MIP/PLL/PEDOT−PG/GCE for the target DBP at the concentration of 100 nM vs. DMP, DEP, DOP, DEHP, and DINP at the concentration of 5 µM. (**D**) Response peak currents of 0.01 pM DBP recorded at six independent MIP/PLL/PEDOT−PG/GCE. (**E**) Six successive determinations of 0.01 pM DBP at the same MIP/PLL/PEDOT−PG/GCE. (**F**) Response peak currents of the sensor in 0.01 pM DBP after 0, 7, and 14 days, respectively.

Under optimal conditions, different concentrations of DBP (1 fM–5 µM) were evaluated by the DPV method by applying the MIP/PLL/PEDOT−PG/GCE, and the results were shown as Figure 6A. A linear relation (Figure 6B) between current response and DBP concentration was in the range of 1 fM–5 µM with an obtained correlation coefficient of 0.9932. The regression equation was ΔI = 10.7275lgC_DBP_ + 199.697, based on which the detection limit (LOD) was estimated to be 0.88 fM. Therefore, compared with other detection methods, this proposed method has a wider detection range and lower LOD, as shown in Table 1. The sensor obtains a wider detection range and a lower detection limit for DBP, which supplies a sensitive detection method for DBP in plastic samples.

#### 3.3.6. Selectivity of the Sensor

It is of great significance to evaluate the selectivity of electrochemical sensors. To check the selectivity of MIP/PLL/PEDOT−PG/GCE, DPV responses of DBP and structural analogs on MIP/PLL/PEDOT−PG/GCE and NIP/PLL/PEDOT−PG/GCE were contrasted, and the results can be seen in Figure 6C. Obviously, the DPV currents difference of the MIP sensor were much larger than that of the NIP sensor, and the DPV response to DBP was higher than that of any other structural analogue. The results indicate that the MIP/PLL/PEDOT−PG/GCE exhibited excellent selectivity toward DBP. Moreover, a value of ratio for each analyte was defined as β (imprint factor), which was calculated as follows: β = ΔI_pa_ (MIP sensor)/ΔI_pa_ (NIP sensor), where ΔI_pa_ is the peak current difference of DBP and structural analogs at 0.178 V in DPV experiments. The results were 14.45, 1.91, 1.33, 2.18, 1.87, and 1.64 for DBP, DMP, DEP, DOP, DEHP, and DINP, respectively. These results indicated that the MIP sensor displayed high recognition selectivity for DBP.

#### 3.3.7. Repeatability, Reproducibility, and Stability of the Electrochemical Sensor

To explore the applicability of the MIP/PLL/PEDOT−PG/GCE, we investigated its repeatability, reproducibility, and stability. Thanks to the electropolymerization method, the membrane thickness can be precisely controlled. Therefore, this also provides the possibility for excellent repeatability of the sensor. To explore the repeatability of the MIP sensor, DPV detection of the same DBP solution was performed using six MIP/PLL/PEDOT−PG/GCE. Based on the obtained current response, the relative standard deviation (RSD) was calculated to be 2.32%, which confirmed that the sensor has dramatic repeatability, shown as Figure 6D. The RSD for six successive determinations of 0.01 pM DBP was 2.13% (Figure 6E), demonstrating the excellent reproducibility of MIP/PLL/PEDOT−PG/GCE. Figure 6F shows the DPV current response of the sensor after 0, 7, and 14 days, respectively, from which it can be observed that the current decreased by 2.5% after 7 days and 6.2% after 14 days, indicating that the current signal of the sensor is basically reliable, and the prepared sensor has favorable stability.

#### 3.3.8. Determination of DBP in Practical Samples

To explore the application of MIP/PLL/PEDOT−PG/GCE in practical samples, DPV detection of DBP concentration in plastic samples was carried out by adding a standard method. The recovery of DBP in several plastic samples was from 97.5% to 106.6%, and the RSD was in the range of 1.4–5.2% (Table 2). Therefore, the results were in preferable agreement with those of the GC−MS method. In summary, MIP/PLL/PEDOT−PG/GCE shows satisfactory accuracy and reliability in the specific recognition and detection of DBP in complicated matrices.

## 4. Conclusions

Based on MIPs and PLL/PEDOT−PG nanocomposites, an ultra-sensitive and label-free electrochemical molecularly imprinted sensor was prepared for the detection of DBP in packaging material samples. PLL/PEDOT−PG improved environmental stabilization and electro catalytic activity, supplying a larger superficial area, which can adsorb more template molecules to the surface, resulting in highly specific recognition sites. Therefore, the prepared MIP/PLL/PEDOT−PG/GCE exhibited a satisfactory linear range (1 fM−5 μM), with extremely low LOD (0.88 fM). In addition, the MIP/PLL/PEDOT−PG/GCE presented excellent selectivity, satisfactory stability, and repeatability, which can realize the rapid and reliable detection of DBP.

## Figures and Tables

**Table 1:** Comparison of analytical approaches used for DBP detection.

Methods	Sample	Linear Range	LOD	References
Fluorescence Polarization Immunoassay	Bottled drinking water	0.5–7.5 μg/mL	0.35 μg/mL (1.25 × 10^−6^ M)	[57]
Colorimetric sensor	Baijiu	0.0–2.8 mg/L	0.05 mg/L (1.79 × 10^−7^ M)	[58]
Electrochemical immunosensor	River water	-	7 ng/mL (2.5 × 10^−8^ M)	[59]
Fluorescence “switch on’’	Water	0.025–1 mM	24 nM (2.4 × 10^−8^ M)	[60]
Fluorescence ratio immunosensor	Liquor	12.5–1500 μg/L	5.0 μg/L (1.79 × 10^−8^ M)	[24]
Molecularly imprinted electrochemical impedance sensor	-	0.01–1.0 μM	4.5 × 10^−9^ M	[61]
Colorimetric immunosensor	Food	-	1 μg/L (3.5 × 10^−9^ M)	[62]
Ratiometric fluorescent immunoassay	Water	2.31–66.84 ng/mL	0.86 ng/mL (3.09 × 10^−9^ M)	[63]
Tricolor ratiometric fluorescence sensor	Seawater	2.0–20.0 × 10^3^ μg/L	0.65 μg/L 2.34 × 10^−9^ M)	[64]
Ratiometric fluorescence ELISA	Water	0.98–73.06 ng/mL	0.17 ng/mL (6 × 10^−10^ M)	[65]
Colorimetric immunoassay	Liquor	150–2700 mg/L (0.54–9.72 mM)	76 ng/L (2.73 × 10^−10^ M)	[66]
MIP electrochemical sensor	Tap water Baijiu	10^−7^ g/L–10^−2^ g/L	5.09 × 10^−9^ g/L (1.83 × 10^−11^ M)	[67]
Electrochemical immunosensor	Liquor	1 pg/mL–0.1 μg/mL	0.276 pg/mL (9.92 × 10^−13^ M)	[23]
MIP electrochemical sensor	Plastic samples	1 fM–5 µM	0.88 fM (8.8 × 10^−14^ M)	This work

**Table 2:** Determination results of DBP in various plastic samples (*n* = 3).

Plastic Samples	Detected (µM)	Added (µM)	DPV			GCMS		
			Found (µM)	Recovery (%)	RSD (%)	Found (µM)	Recovery (%)	RSD (%)
PVC1-W	ND	0.05	0.0512	102.4	2.9	0.0495	99.0	4.7
		0.5	0.4963	99.3	4.5	0.5113	102.3	3.8
PVC1-C	ND	0.05	0.0494	98.8	4.8	0.0512	102.4	1.4
		0.5	0.5102	102.0	5.3	0.4962	99.2	4.7
PVC2-W	ND	0.05	0.0489	97.8	3.7	0.0485	97.0	5.2
		0.5	0.5226	104.5	2.6	0.4936	98.7	3.3
PVC2-C	ND	0.05	0.0492	98.5	2.7	0.0490	98.0	3.2
		0.5	0.4933	98.7	6.3	0.4926	98.5	3.7
PET-W	ND	0.05	0.0533	106.6	3.8	0.0522	104.4	3.6
		0.5	0.4896	97.9	5.4	0.5205	104.1	2.9
PET-C	ND	0.05	0.0511	102.2	5.6	0.0511	102.2	4.8
		0.5	0.4875	97.5	2.8	0.4976	99.5	2.9

ND: Not detected.

## Data Availability

Data are contained within the article.
